# Editorial: Socio-Psychological Influences on Pain

**DOI:** 10.3389/fpain.2022.1122466

**Published:** 2023-01-09

**Authors:** Peter D. Drummond, Melinda Nicola

**Affiliations:** School of Psychology and Centre for Healthy Ageing, College of Health and Education, Murdoch University, Murdoch, WA, Australia

**Keywords:** pain invalidation, stigma and discrimination, adolescence and young adulthood, pain in children, gender identity and pain

**Editorial on the Research Topic** Socio-Psychological Influences on Pain

## Introduction

The collection of papers on Socio-Psychological Influences on Pain deepens the conversation around the psychosocial factors that contribute to pain-related experiences. A recurring theme throughout the Research Topic is the issue of pain-invalidation. Within the broader chronic pain literature, invalidation of pain has been linked with stigma due to a lack of belief, understanding and compassion by healthcare providers and/or close friends and family of people in pain (1). An additional theme, relating to a reluctance to accept one's own pain or of failing to live up to one's core values and beliefs, may result in guilt and shame about burdening others. This critical self-judgement increases the risk of social isolation and mood disturbances such as anxiety and depression. Invalidation of pain reflects unacceptability of an individual's expression of pain and implies withdrawal of social support. This lack of social acceptance represents a stress on the individual's resources, adding to the burden of chronic pain.

Within this Research Topic, these component themes form the framework for a definition of pain-invalidation that incorporates three essential elements: failing to believe that the experience of chronic pain is true for the individual sufferer, effectively delegitimizing their experience; failure to acknowledge that the individual's pain is acceptable; and failing to communicate to the sufferer that their pain is real and acceptable (Nicola et al.).

Kudrina and colleagues emphasize the issue of pain-invalidation in adolescents and young adults, with the age group (16–25 years) remaining largely unseen in terms of their pain experience. People within this age group are particularly susceptible to risk factors such as drug experimentation, psychiatric illness, and suicide. Kudrina et al. propose the need for specialized training of healthcare professionals, as well as resources tailored to facilitate communication and support for this transitional group.

Indeed, age in relation to the pain experience was central to the Research Topic, with several articles suggesting that the best-suited approach toward individuals must consider the cognitive capacities of their age group. For example, in a thematic analysis by Pavlova et al. regarding parent's beliefs on reminiscing about past pain experiences with their children, parents with younger children were more inclined to avoid reminiscing to process past pain than parents of older children. Some parents, instead, felt the need to protect their child, preferring to avoid eliciting negative emotions related to pain, while others were more inclined to reminisce about pain experiences as a learning tool and to emotionally process past pain. The authors discuss the benefits of using suitable language and context in conversations with children to promote understanding of their own pain and empathy for others.

The importance of providing a language for understanding pain was a shared theme across several articles. In another paper by Pavlova and colleagues, parents' beliefs about how pain is depicted in children's media were explored. Depictions of pain were seen by parents as being used either to entertain and amuse children or to convey valuable lessons. Portrayals of pain in the media serve as a general model and provide young children with the language to understand and express their own pain experiences. Parents may use these portrayals as an opportunity to talk with their child about unrealistic representations and to suggest more appropriate emotional responses and empathic reactions. In addition, Pavlova et al. noted parents' comments that male characters experienced more physical pain and were more resilient to pain than female characters, while female characters tended to face more emotional aspects of pain and were more likely to ask for, and receive, help. The authors raise the question of how such portrayals may impact on potential gender differences in pain.

The issue of gender differences was also discussed in relation to reporting adverse effects of pain medication. Nguena Nguefack and colleagues studied people with chronic pain aiming (i) to discover the main non-life-threatening, adverse effects of pain medications, and (ii) to identify individuals most likely to report these adverse effects. Adverse effects of pain medication were associated both with gender identity and gender roles. Women were more likely than men to report severe adverse effects from pain medications; additionally, though, participants with masculine or androgenous characteristics were more likely to report severe adverse effects than participants with undifferentiated or feminine characteristics. Bearing this in mind, information and consultation should be tailored to help patients make informed decisions regarding their selection of pain medications.

In sum, the papers in this Research Topic draw attention to important psychosocial influences on the experience and management of chronic pain and emphasize the worth of including these factors within a comprehensive biopsychosocial framework. Central to this approach is acknowledging and validating the patient's lived experience of pain, and recognizing that individual differences (e.g., in cognitive development, age, gender roles and gender identity) uniquely influence each person's pain experience. Validation of pain normalizes this experience and creates opportunities for emotional processing. Crucially, pain validation also increases adherence to treatment protocols and the likelihood of treatment success Nicola et al. Thus, listening to the patient nonjudgmentally and validating their experience of pain are key first steps in moving the treatment process forward.
